# High-speed, high-frequency ultrasound, *in utero* vector-flow imaging of mouse embryos

**DOI:** 10.1038/s41598-017-16933-x

**Published:** 2017-11-30

**Authors:** Jeffrey A. Ketterling, Orlando Aristizábal, Billy Y. S. Yiu, Daniel H. Turnbull, Colin K. L. Phoon, Alfred C. H. Yu, Ronald H. Silverman

**Affiliations:** 1 Frederic Lizzi Center for Biomedical Engineering, Riverside Research, New York, NY 10038 USA; 2 Skirball Institute of Biomolecular Medicine and the Department of Radiology, New York University School of Medicine, New York, NY 10016 USA; 3 Department of Electrical and Computer Engineering, University of Waterloo, Waterloo, ON N2L3G1 Canada; 4 Hassenfeld Children’s Hospital at New York University Langone, New York, NY 10016 USA; 5 Department of Ophthalmology, Harkness Eye Institute, Columbia University Medical Center, New York, NY 10032 USA

## Abstract

Real-time imaging of the embryonic murine cardiovascular system is challenging due to the small size of the mouse embryo and rapid heart rate. High-frequency, linear-array ultrasound systems designed for small-animal imaging provide high-frame-rate and Doppler modes but are limited in regards to the field of view that can be imaged at fine-temporal and -spatial resolution. Here, a plane-wave imaging method was used to obtain high-speed image data from *in utero* mouse embryos and multi-angle, vector-flow algorithms were applied to the data to provide information on blood flow patterns in major organs. An 18-MHz linear array was used to acquire plane-wave data at absolute frame rates ≥10 kHz using a set of fixed transmission angles. After beamforming, vector-flow processing and image compounding, effective frame rates were on the order of 2 kHz. Data were acquired from the embryonic liver, heart and umbilical cord. Vector-flow results clearly revealed the complex nature of blood-flow patterns in the embryo with fine-temporal and -spatial resolution.

## Introduction

The mouse is the most common experimental organism used to study gene function and cardiovascular disease (CVD)^1^. The heart is the first organ to function in the mammalian embryo and, together with the developing vasculature, is critical to the delivery of oxygen and nutrients to embryonic tissues. While zebrafish and chick embryos are often used for cardiovascular development studies^2^, the mammalian mouse is the model of choice to study CVD^1^, including heart failure^3^, valvular disease^4^, and congenital heart defects^5^.

The interrelation of cardiac mechanics and blood flow patterns is important for understanding normal and abnormal cardiac function^6,7^ as well as the development and maturation of the heart^8,9^ particularly in the context of sophisticated computational fluid dynamics modeling^10,11^. Features of flow such as vortex patterns in the ventricle have been proposed as an indicator of cardiac health^12,13^. State-of-the-art cardiovascular imaging of the human heart has yielded insights into aspects of this complexity of normal cardiac function and blood flow^14,15^ as well as pathophysiological mechanisms in a variety of CVDs such as cardiomyopathies and aortopathies^6,13^.

Measurements of cardiovascular mechanics and hemodynamics in embryonic mice are challenging because of the small size (10 to 15 mm crown-rump length) and rapid heart rate (200–250 bpm embryonic and >400 bpm at one week of age; anesthetized^16^). The developing embryo is particularly sensitive to disruptions in the cardiovascular system and *in utero* lethality often results in transgenic mice with a cardiac phenotype^17^. High-frequency ultrasound (HFU above ≈20 MHz) has emerged as an important imaging modality for small animals and it is currently the only viable imaging modality for real-time *in utero* cardiac and flow imaging in the mouse embryo.

Early HFU Doppler measurements were based on single-element transducers and allowed a small sample volume to be interrogated but only permitted blood velocity spectrograms at one spatial location at a time over a limited depth of field^18^. Commercial HFU scanners based on linear-array transducers (i.e., VisualSonics, FujiFilm, Toronto, Canada) are able to provide excellent image resolution with center frequencies >30 MHz^19^. These systems have standard Doppler imaging modes, such as color-flow and power Doppler, and have been used to characterize *in utero* cardiac function in embryonic mice^20^. However, Doppler information cannot be obtained with simultaneous fine-temporal and -spatial resolution because traditional Doppler implements a line-by-line based method of transmission. In addition, traditional Doppler approaches are only able to estimate the axial component of flow velocity, relative to the probe, and are unable to depict complex flow patterns. Thus, it is difficult to assess whether the hemodynamics of mouse models fully mirrors the human case in terms of blood flow profiles and patterns. Ideally, imaging of CVD mouse models would have the same sophistication as approaches being pursued for humans so that insight from mouse models could be directly translated to human CVD^21^.

The limits of traditional Doppler ultrasound for assessing hemodynamics can be overcome by implementing high-speed, plane-wave imaging^22^ and multi-angle vector-flow techniques^23^. The basic concept is to rapidly transmit, at rate *R*, wide-field-of-view plane waves into tissue, by firing all of the elements of a linear array with inter-element transmission delays that create a coherent wave front at a steered angle. The return echoes from each transmission are then digitized on all of the array elements and delay-and-sum beamforming is implemented to generate a full 2D image from each transmission. By coherently compounding (e.g., summing) beamformed data acquired with a set of *M* steered transmit angles, the signal-to-noise ratio and resolution can be improved while still maintaining a high transmission rate, although compounding will reduce the final, effective frame rate to *R*/*M* ^24^. An additional benefit of *M* transmission angles is that multi-angle, vector-flow methods can be applied in order to yield axial and lateral velocity estimates at each slow-time pixel without any assumptions regarding the angle of flow relative to the probe. This is in contrast to traditional Doppler that only provides an estimate of axial velocity and requires a Doppler angle to be defined in order to make an estimate of non-axial flow. Traditional Doppler cannot resolve lateral flow nor can it provide information on flow patterns.

The absolute rate of transmission, *R*, is limited by the round trip acoustic propagation time in tissue. As imaging depth increases, maximum frame rate decreases. For a 2-cm imaging depth, a 37-kHz frame rate can be sustained whereas a 4-cm depth would reduce the maximum frame rate to 18.5 kHz. The transmission rate also has an impact on the Doppler aliasing cut off which is inversely proportional to the transducer center frequency and proportional to the frame rate (i.e., inverse of slow-time sampling interval). Keeping all parameters the same, a ten times increase in transducer frequency lowers the peak detectable axial velocity by a factor of ten while a ten times increase in frame rate increases the peak detectable velocity by a factor of ten. In the context of HFU, the shallow penetration depths allow for high values of *R*.

While vector-flow plane-wave imaging methods have been applied to cardiac and blood-flow studies at lower frequencies in human adults^25^ and newborns^26^, the methods have yet to be adapted to the specific requirements of HFU mouse-embryo imaging. Here, we demonstrate the feasibility of *in utero* vector-flow imaging of blood flow in mouse embryos using a high-speed, HFU plane-wave and vector-flow imaging approach capable of sub-ms, full-frame image capture.

## Results

### Rotational Phantom

The data acquisition and processing methods were validated using a 1-cm diameter rotational scattering phantom. Steered ultrasound plane waves were transmitted at an absolute frame rate of 12.5-kHz with *M* = 5 transmission angles spanning ±10 degrees where 0 degrees is normal to the transducer face. The acquired data were beamformed and each set of *M* = 5 transmission angles were compounded before generating log-compressed grayscale B-mode images. The effective frame rate of the image sequence after compounding was 2.5 kHz which represented the slow-time Doppler sampling rate. Based on a transmit center frequency of 17.9 MHz and the slow-time sampling rate of 2.5 kHz, the maximum resolvable Doppler velocity at the Nyquist limit, before aliasing, was 5.3 cm/s. The phantom rotated at 1 rev/s or, in terms of velocity at the outermost edge, 3.1 cm/s. The uncompounded beamformed data were processed in a separate pipeline to obtain multi-angle vector-flow estimates.

**Figure 1:**
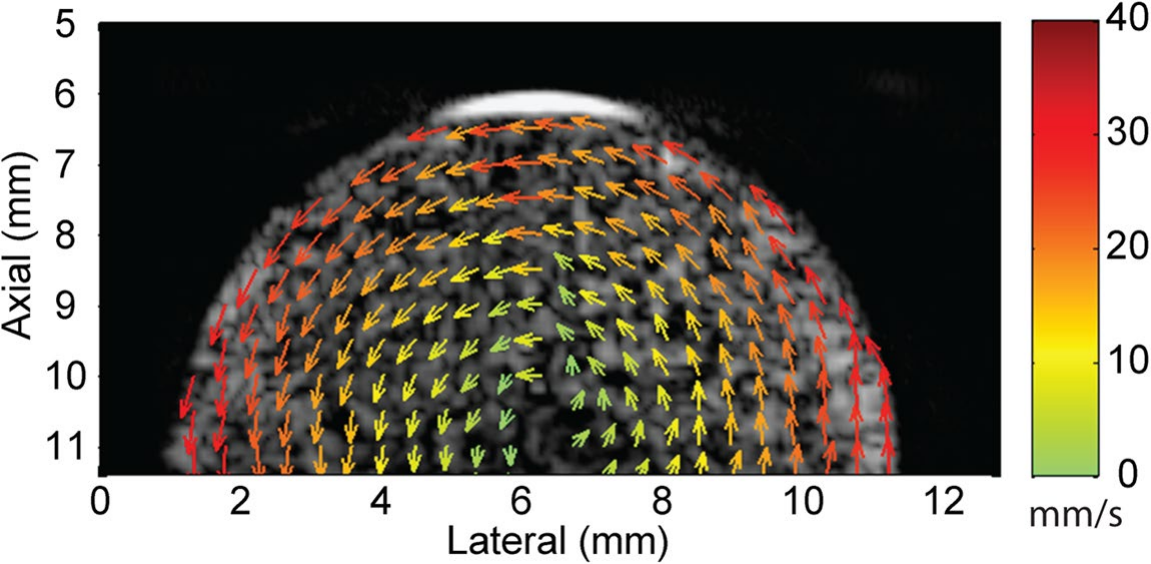
Vector-flow map of a 1-cm diameter scattering phantom rotating counter-clockwise with a maximum expected velocity of 3.1 cm/s at the outer edge of the phantom. The vector-flow map indicates the direction of rotation with a velocity gradient from the maximum velocity at the outer edge to zero in the center of the phantom. Unlike when using traditional Doppler, lateral flow components could be resolved.

Figure 1 shows the vector-flow estimates throughout the phantom with the elevation focus of the array placed two millimeters below the leading edge of the phantom. The vector-flow information revealed the counter-clockwise rotation of the phantom with zero velocity at the center of the phantom and 3.1 cm/s at the outer edge of the phantom. No assumptions were made regarding the Doppler angle and, unlike with traditional Doppler, lateral flow components could be resolved.

**Figure 2:**
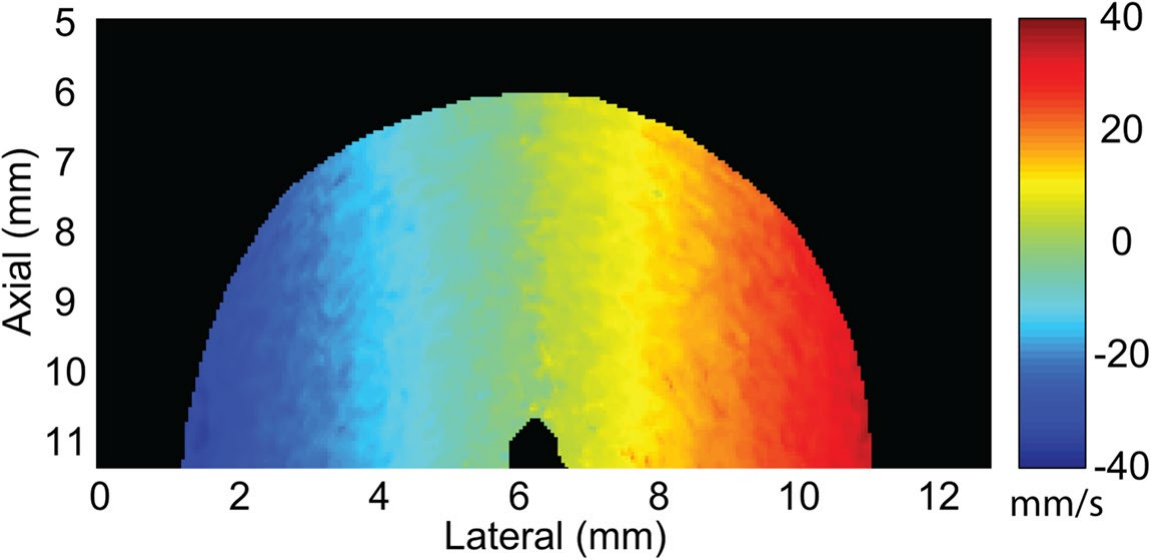
Color-flow Doppler axial velocity map of a 1-cm diameter scattering phantom rotating with a maximum expected velocity of 3.1 cm/s at the outer edge of the phantom. The peak velocities on either side of the phantom, where the Doppler angle was 0 degrees, agreed with the expected maximum velocity. The central region of the phantom where the axial motion was zero represents a blind zone when using traditional Doppler methods.

The multi-angle, vector-flow method of Doppler analysis can be compared to traditional color-flow Doppler estimates calculated from the same phantom data after compounding each ensemble batch of *M* = 5 transmit angles (Fig. 2). The velocities span the range of ±3.1 cm/s with the central portion of the phantom having zero velocity, as would be expected for particles with no axial velocity component. The outermost edges of the phantom at 0 and 10 mm lateral location had only an axial velocity component and agreed with the maximum expected velocity.

### Mouse Embryo

The high-speed image acquisition and vector-flow algorithms were applied to data acquired from *in utero*, E16.5 (where E0.5 was defined as noon of the day after successful overnight mating), mouse embryos in order to emphasize the blood circulation in the developing cardiovascular system. Data were acquired with the mother anesthetized, resting supine on an imaging platform and with the stomach shaved.

**Figure 3:**
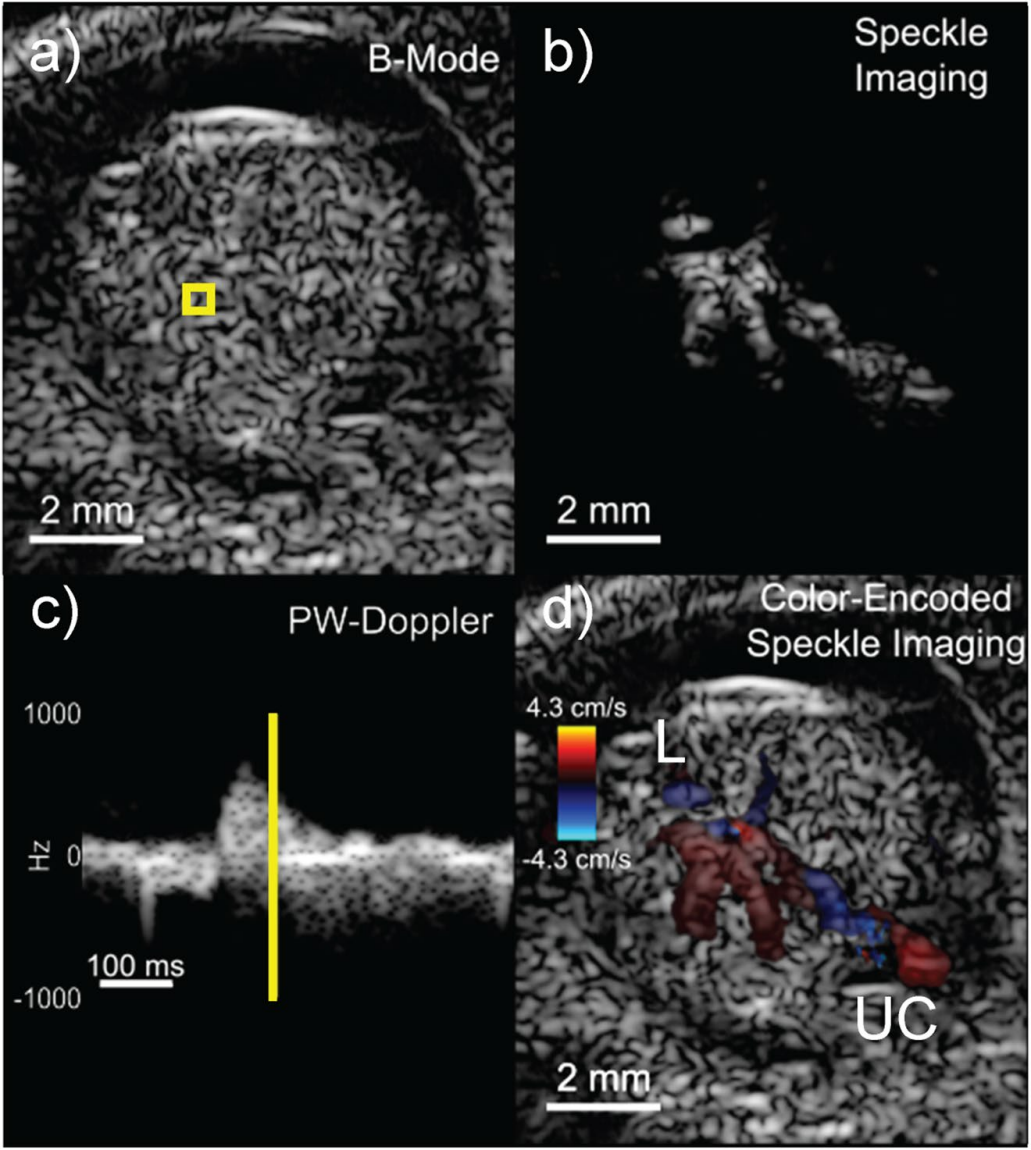
One frame of 515-ms duration, high-speed acquisition from an E16.5 embryo with flow in the liver (L) and umbilical cord (UC) showing (**a**) B-mode image, (**b**) non-stationary flow speckle, (**c**) Doppler spectrogram in the region of the liver using the averaged slow-time data from the boxed region in (**a**), **d**) color-flow Doppler image. The yellow line in (**c**) 258 ms after the start of the spectrogram, indicates the slow-time point of the images. The full image sequence is available as Movie 1.

Data were acquired from an image plane containing the liver and umbilical cord using a 10-kHz absolute frame rate with *M* = 5 transmission angles spanning ±10 degrees which yielded an effective slow-time frame rate of 2 kHz after beamforming and compounding. The maximum possible axial flow velocity at the Nyquist limit, before aliasing, was 4.3 cm/s. Figure 3 shows a single frame from a 515-ms duration high-speed image sequence (Movie 1) obtained from the same E16.5 embryo. The data are presented as a) a standard log-compressed grayscale image, b) a flow-speckle map after clutter filtering, c) a Doppler spectrogram from a region of interest (ROI) and d) a color-encoded speckle image overlaid on the B-mode image. Even with the limited anatomical resolution of the 18-MHz transducer, a shifting speckle pattern from blood flow was clearly observed in the image sequence (Fig. 3b).

The high-speed movie sequence spans just over one cardiac cycle. The image represents a time point (258 ms from start of spectrogram) in the systolic phase when blood was moving out of the embryo through the umbilical artery (blue region of UC in Fig. 3d) and through the highly perfused liver. The umbilical vein (red region of UC in Fig. 3d) revealed an undulating, continuous flow into the embryo. This was consistent with past observations of umbilical venous flow with very mild pulsatility in one direction^27,28^. The large branches of flow represent the lobes of the liver which are fully defined at 16.5 days^29^.

**Figure 4:**
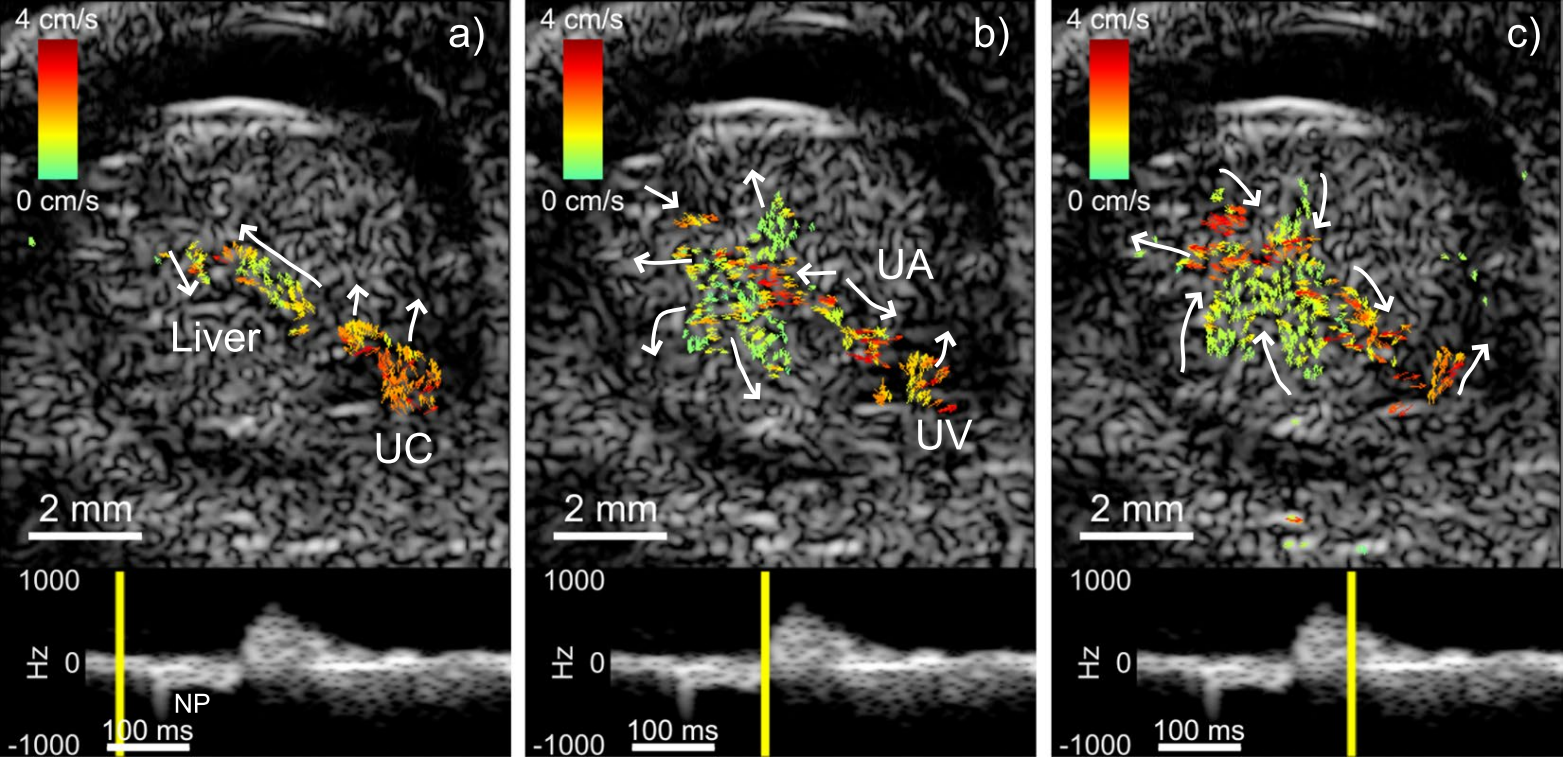
Three frames (40, 186 and 258 ms relative to start of spectrogram) from the vector flow analysis of the data sequence of Fig. 3. The Doppler spectrogram represents the same region of the liver as was indicated in Fig. 3a. The yellow line in the spectrogram indicates the slow-time point for which the data is displayed. (**a**) Diastole where the perfusion in the liver was minimal. (**b**) Late diastolic phase with a reversal of flow. (**c**) Systolic phase after the spectrogram peaked in the positive flow direction. The white arrows highlight the general direction of local flow vectors in each frame. Flow vectors point in the direction of flow and their color and length scale with the vector magnitude. NP: Negative pulse 93 ms after start of spectrogram, UA: Umbilical artery, UV: Umbilical vein. The full image sequence is available as Movie 2.

The vector-flow analysis of the Fig. 3 data sequence revealed a more detailed behavior of the blood flow in the embryo. Figure 4a represents a point in diastole when the perfusion in the liver was minimal. At late diastole, a negative Doppler pulse was visible 93 ms after the start of the spectrogram, possibly due to atrial contraction, and the flow was temporarily reversed (Fig. 4b). During systole, the vector flow changed direction, as was also seen in the spectrogram (Fig. 4c). Note that there were flow vectors in the lateral direction. Traditional color-flow imaging would not resolve these components of the flow.

**Figure 5:**
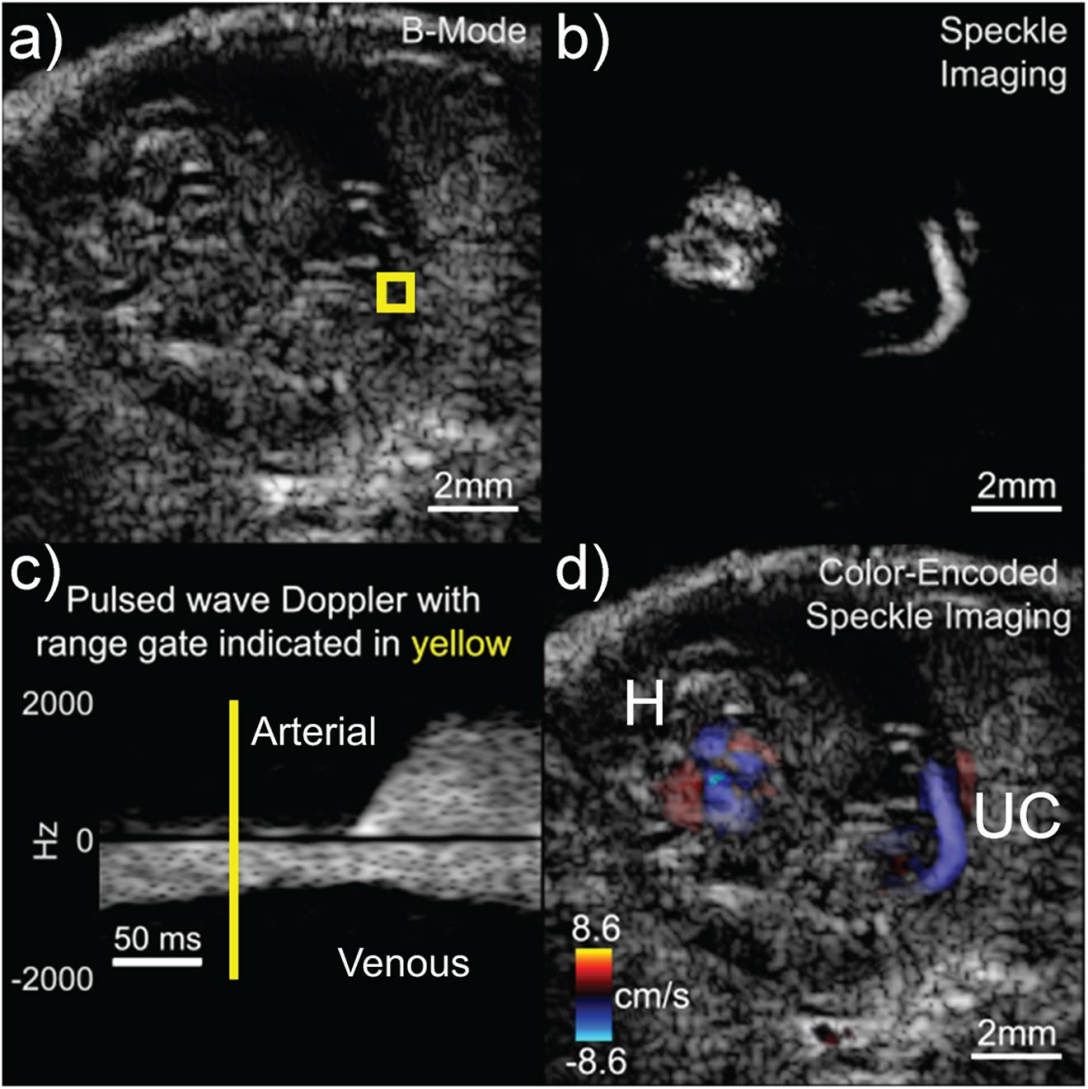
One frame of a 245-ms duration, high-speed acquisition from an E16.5 embryo in the area of the heart (H) and umbilical cord (UC) showing (**a**) B-mode image, (**b**) non-stationary flow speckle, (**c**) Doppler spectrogram in the region of the UC using the averaged slow-time data from the boxed region in (**a**), **d**) color-flow Doppler image. The yellow line in (**c**) 76 ms after the start indicates the time point of the images. The full image sequence is available as Movie 3.

With the imaging plane showing the embryonic heart and umbilical vessels, data were acquired using an absolute frame rate of 20-kHz with *M* = 5 transmission angles spanning ±10 degrees which yielded an effective slow-time frame rate of 4 kHz after beamforming and compounding. The frame rate was increased to 20 kHz for this intracardiac case to reduce the chance of aliasing. Figure 5 shows a single frame from a 245-ms duration high-speed image sequence (Movie 3). The maximum possible axial flow velocity at the Nyquist limit, before aliasing, was 8.6 cm/s. The high-speed movie sequence spans just under one cardiac cycle and the expansion and contraction of the heart can be observed. Figure 5c shows a Doppler spectrogram derived from a region of the umbilical cord indicated by the yellow box in Fig. 5a. The spectrogram revealed the expected pulsatility in the arterial flow and the more continuous flow in the venous vessels^27,28^. With color-flow Doppler flow information overlaid on top of the B-mode, the umbilical artery (red), umbilical vein (blue) and heart were well visualized (Fig. 5d).

**Figure 6:**
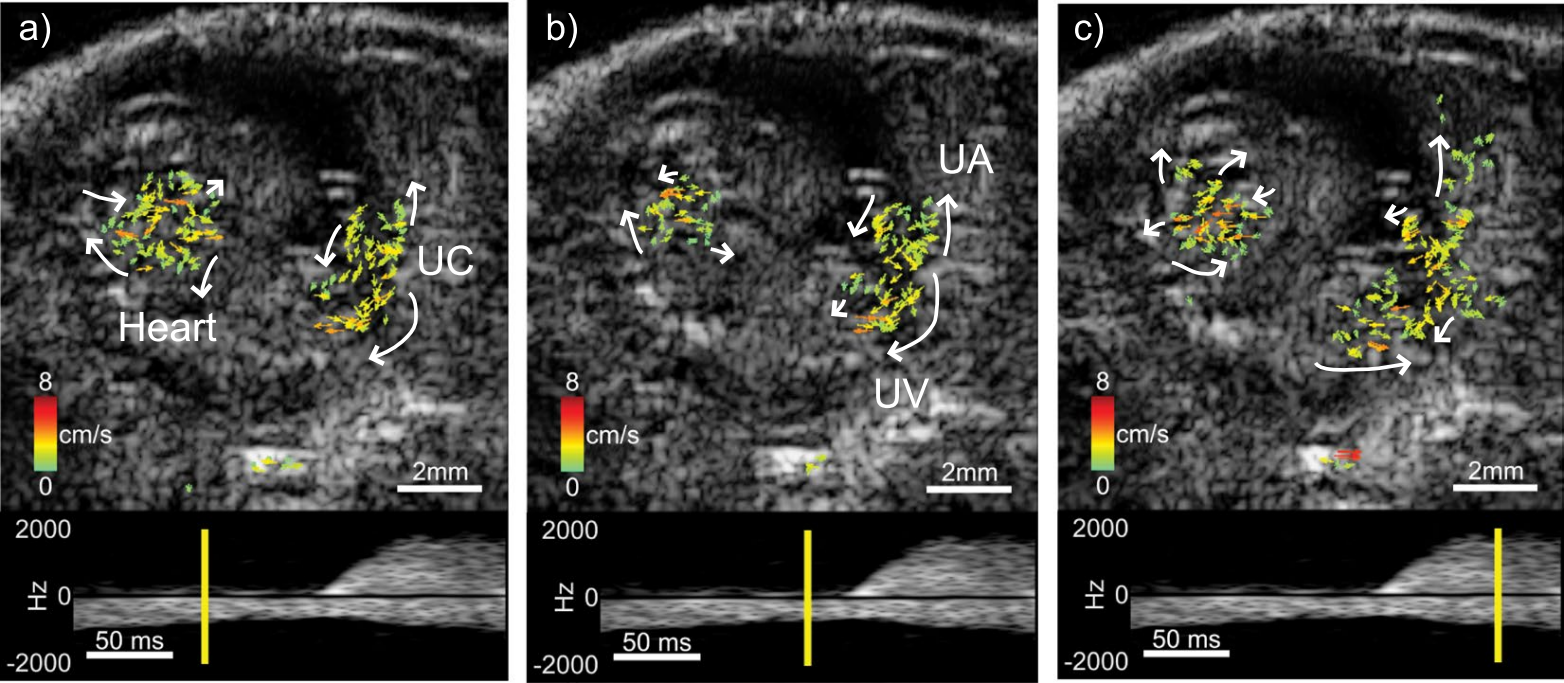
Three frames (76, 119 and 212 ms relative to start of spectrogram) from the vector-flow analysis of the data sequence of Fig. 5. The Doppler spectrogram represents the same region of the umbilical cord (UC) as was indicated in Fig. 5a. The yellow line in the spectrogram indicates the slow-time point for which the data is displayed. (**a**) Early systole with steady flow in umbilical vein. (**b**) Late diastole to early systole where flow in heart is minimal. (**c**) Flow in heart increasing during diastole. The white arrows highlight the general direction of local flow vectors in each frame. Flow vectors point in the direction of flow and their color and length scale with the vector magnitude. UA: Umbilical artery, UV: Umbilical vein. The full image sequence is available as Movie 4.

The vector-flow analysis of the Fig. 5 data sequence revealed the complex nature of the blood flow patterns in the embryo (Fig. 6). Figure 6a shows the early systolic phase of the cardiac cycle just before contraction with a continuous flow in the umbilical vein. Figure 6b shows late diastole to early systole where flow in the heart was minimal and umbilical vein flow remained steady. Figure 6c shows the flow in the heart chambers while the umbilical artery revealed an increased flow after the earlier heart contraction in Fig. 6b.

## Discussion

The multi-angle vector-flow approach used here has the advantage of providing quantitative estimates of blood flow patterns that are not dependent upon the orientation of the probe relative to the flow^23^. Traditional Doppler methods typically rely on a fixed angle of transmission and the Doppler signal is obtained from the axial component of the flow, but an accurate estimate of flow velocity can only be obtained if an assumption is made on the direction of flow relative to the probe. For this reason, a power-Doppler display is often used to indicate where there is flow without concern about velocity. The multi-angle vector-flow approach to Doppler estimates allows for directional and magnitude estimation based on a series of transmission angles that are fixed relative to the probe. As few as three transmit angles spanning ±10 degrees were found to yield good velocity estimates^23^.

Embryonic blood velocities using traditional Doppler methods are typically reported as peak velocities obtained from spectrograms. Representative velocity values from the literature that are relevant to our measurements are time averaged maximum umbilical vein flow of 5.5 cm/s at E17.5^20^; peak umbilical artery flow of 10 cm/s and peak umbilical vein flow of 4.5 cm/s at E16.5^16^; and mitral and tricuspid A wave flows of 40 cm/s and 38 cm/s, respectively, at E17.5^30^. Velocity distributions within cardiac chambers are not reported because measurements are typically made where a flow direction can be defined. Although we expected to see some regions where peak velocities exceeded the Doppler aliasing cutoff, the lag-one autocorrelation approach provided mean values that were below the aliasing limit and the observed velocity ranges we saw in the heart, liver and umbilical cord were consistent with values reported in the literature.

One limitation of vector-flow imaging and HFU is Doppler aliasing. With flow in one direction, the Doppler angle can simply be changed to reduce the axial velocity component relative to the probe and avoid aliasing, but when local flow is in many directions (e.g., a vortex), changing the Doppler angle will not be of much use. In addition, because the aliasing cut-off is inversely proportional to transducer frequency, the use of HFU inherently reduces the maximum velocity before aliasing. Therefore, advanced dealiasing algorithms may be necessary in cases where the flow velocities are expected to be high, particularly for intracardiac flow in post-natal mice. For instance, peak mitral flow in two week old mice can reach 60 cm/s^30^ and peak aortic flow velocities in three week old mice can reach 75 cm/s^16^.

For our plane-wave studies, we used an 18-MHz linear array with an ultrasound platform designed for research. The sample volume of the transducer was 0.3 × 0.4 × 0.13 mm in the lateral, elevation and axial directions, respectively. Typically, embryonic imaging is performed with transducers having center frequencies in the 30 to 40 MHz range which would improve resolution by a factor of roughly 1.5 or 2, respectively. However, Doppler measurements are often performed at frequencies below what would be considered the optimal B-mode transducer frequency because the lower frequency undergoes less attenuation from acoustic propagation and increases the Doppler aliasing cut-off. Our ultrasound system had a maximum sampling rate of 62.5 MHz and, therefore, operating much beyond 20 MHz with our transducer would have sacrificed transducer bandwidth, reduced sensitivity and required working near the Nyquist sampling limit.

## Conclusion

For the first time, vector-flow information has been obtained from the cardiovascular system of *in utero* mouse embryos using HFU and high-speed, plane-wave imaging techniques. Numerous studies have demonstrated the methods in humans, but the transducers and instrumentation necessary to translate the methods to small animals are not yet widely available. By processing the plane-wave data obtained from the embryos with a multi-angle vector-flow approach, we were able to obtain vector-based blood-flow patterns revealing detailed information related to flow in the heart, liver and umbilical cord. These initial results indicate that plane-wave imaging and multi-angle vector-flow processing have the potential to be a useful tool to study cardiovascular function and disease in mouse embryos.

## Methods

### Data Acquisition

High-speed plane-wave data were acquired with a Vantage 128 (Verasonics, Redmond, WA) and an 18-MHz, 128 element linear array with an 8-mm elevation focus (Verasonics L22-14v). Plane waves were transmitted at absolute frame rates, *R*, between 10 and 20-kHz with sets of *M* = 5 transmit angles spanning ±10 degrees with equal spacing between angles (i.e., −10, 5, 0, 5, 10) where zero degrees was normal to the array surface. For each transmit event, all 128 array channels were excited and the receive echoes were digitized on all 128 channels. The transmit frequency was 17.9 MHz and the duration of the transmit waveform was 1.5 cycles. The −6-dB lateral resolution of the probe was ≈0.15 mm, the slice thickness at the elevation focus was ≈0.9 mm and the axial resolution was 0.14 mm. A duplex Doppler mode consisting of a real-time B-mode image with a color-flow Doppler overlay was used to locate an ROI. Once an ROI was selected, a high-speed plane-wave acquisition was initiated. After all of the transmissions, pre-beamformed channel data were streamed to the host PC and the system was returned to the duplex mode in preparation for the next acquisition.

### Rotational Phantom

A rotational scattering phantom was employed to validate that the flow estimation algorithms generated accurate velocity estimates under controlled conditions. The same imaging protocols used for the mouse imaging were used for the phantom study. A 1-cm diameter, cylindrical, tissue-mimicking-gel phantom (ATS, Bridgeport, CT) was attached to a DC-servo motor and rotated at a constant angular velocity. The phantom contained scattering particles and was rotated in the plane of the linear array such that the 1-cm diameter, circular cross section was revealed in the image. The phantom provided a simple means of generating a full, continuous range of velocity magnitudes and directions in a single data set. For these experiments, the probe was clamped to a table and held in a fixed position relative to the phantom.

### Animals

The mice used in these studies were maintained under protocols approved by the Institutional Animal Care and Use Committee at New York University School of Medicine. All experiments were performed in accordance with relevant guidelines and regulations. The embryos of timed pregnant Swiss Webster mice (Taconic, Hudson, NY, USA) were imaged at day E16.5, where E0.5 was defined as noon of the day after successful overnight mating. The adult mice were anesthetized with an intraperitoneal injection of ketamine/xylazine with a dose of 75/25 mg/kg of body mass and placed in a supine position on an imaging stage with each limb taped to the stage. The mice were kept at a physiologic temperature of 37 °C using a flow of warm air and the temperature was monitored via rectal thermometer. Before imaging, the abdomen of the mother was shaved and a gel was applied in order to provide acoustic coupling for the ultrasound probe. The probe was held free hand for these experiments.

### Beamforming

The data were beamformed in post processing using a graphic-processing-unit- (GPU) based algorithm that generated in-phase (I) and quadrature (Q) signal components^31^. The GPU device used in this work was the GTX-1080 model (NVIDIA Corp., Santa Clara, CA), and the GPU codec was compiled as a MATLAB (The MathWorks Inc., Natick, MA) executable (MEX) file whose calls could be invoked through programmable scripts in MATLAB. The beamforming algorithm worked as follows: First, raw channel data corresponding to one transmit event with a particular steering angle were converted to a complex analytic form through a Hilbert transform operation. Second, the analytic forms of the channel data were beamformed at each pixel position within an image frame using a delay-and-sum process that accounted for two-way propagation delays between a pixel position and each of the 128 array elements. The beamforming delays could be readily adjusted to assume different receive steering angles. The pixel dimension of the beamformed images was 50 × 50 *μ*m.

### Image Visualization and Flow Estimation

Beamformed data were used to obtain images and flow estimates using a traditional-Doppler or a multi-angle vector-flow approach. For the traditional approach, the sets of *M* transmit angles were beamformed with the receive steering angle set to be the same as the transmit angle and then coherently compounded. The compounded data were then used to generate log-compressed, grayscale B-mode images; flow-speckle maps; Doppler spectrograms; and color-flow maps.

Flow-speckle maps were derived as masked B-mode image frames that only showed the grayscale map value at pixels with inter-frame decorrelation (pixel blackout was applied otherwise). The maps were obtained after using a slow-time processing algorithm that entailed clutter filtering^32^. An equiripple, highpass, finite impulse response clutter filter of order 134 was first applied to the slow-time ensemble of each pixel position to isolate pixels with inter-frame decorrelation^33,34^. A normalized cutoff frequency of 0.1 (i.e., 400 Hz for a 4 kHz slow-time sampling rate) with 100 dB attenuation was selected based on empirical observations of filtering efficacy in terms of background clutter to blood Doppler spectrum. Tissue motion artifacts from movement of the embryo or ultrasound probe were very minimal ($\ll$0.1 cm/s) relative to the expected flow velocities.

Doppler spectrograms were obtained from the filtered, flow-speckle maps. First, compounded frames were stacked to form a 3-D data array (with two spatial dimensions and a slow-time dimension), and the corresponding slow-time ensembles for pixel positions within a specified ROI were extracted. After that, the means of all pixels within the ROI were obtained at each slow-time sampling instant. Finally, the short-time Fourier transform was used to compute a spectrogram of the nominal slow-time ensemble (sliding window size of 64; sliding step size of 2).

To derive color-flow maps, further processing was performed on the filtered slow-time data. A lag-one autocorrelation phase estimation algorithm^35^ was used to estimate the instantaneous slow-time mean frequency using the same parameters as those used for the spectrogram calculation. The mean-frequency estimates were then converted into velocity values using the Doppler equation, and the results were color coded using a red-blue color hue for visualization and overlaid on the grayscale B-mode image.

Vector-flow maps were calculated using a multi-angle Doppler estimation strategy^36^. For each of the *M* transmit angles, beamformed data frames were derived for *M* receive steering angles where the *M* receive angles were the same as the *M* transmit angles. Thus, *M* × *M* unique transmit-to-receive angle pairs were generated and the effective frame rate was *R*/*M*. Subsequently, pixel-wise slow-time clutter filtering and instantaneous slow-time frequency estimation were performed over each transmit-to-receive angle pair using the same sliding window parameters employed for the color-flow mapping. The *M* × *M* frequency estimates at each pixel position and at each slow-time sampling instant, were then used for vector-flow estimation by applying a least-squares fitting process^23^. The derived flow vectors were visualized as color-coded arrows, overlaid on a compounded B-mode image, whose pointing direction corresponded to the estimated vector angle, and whose arrow length and color were pegged to the vector magnitude.

## Electronic supplementary material

B-mode and color-flow Doppler in liver and umbilical cord.

Vector flow in liver and umbilical cord.

B-mode and color-flow Doppler in heart and umbilical cord.

Vector flow in heart and umbilical cord.
